# Crystal structure of 3,6-bis­(pyridin-2-yl)-1,4-di­hydro-1,2,4,5-tetra­zine

**DOI:** 10.1107/S205698901801753X

**Published:** 2019-01-01

**Authors:** Kinga Wzgarda-Raj, Agnieszka J. Rybarczyk-Pirek, Sławomir Wojtulewski, Marcin Palusiak

**Affiliations:** a Group of Theoretical and Structural Chemistry, Department of Physical Chemistry, Faculty of Chemistry, University of Łódź, Pomorska 163/165, 90-236, Łódź, Poland; b Department of Theoretical Chemistry, University of Białystok, Ciołkowskiego, 1K, 15-245 Białystok, Poland

**Keywords:** crystal structure, 1,2,4,5-tetra­zine, hydrogen bond

## Abstract

The structure of the title compound, C_12_H_10_N_6_, at 100 K has monoclinic (*P*2_1_/*n*) symmetry. Crystals were obtained as a yellow solid by reduction of 3,6-bis­(pyridin-2-yl)-1,2,4,5-tetra­zine. The structure displays inter­molecular hydrogen bonding of the N—H⋯N type, ordering mol­ecules into infinite ribbons extending along the [100] direction.

## Abstract 2

In the crystal structure, inter­molecular N—H⋯N hydrogen bonds link the mol­ecules into infinite ribbons extending along the [100] direction.

## Chemical context   

*s*-Tetra­zines represent a class of heterocyclic compounds. The substitution of four nitro­gen atoms in a six-membered benzene-like ring results in strong π-electron deficiency and concentration of negative charge on the heteroatoms. As a result of these properties, *s*-tetra­zines are used in organic synthesis (Saracoglu, 2007 ▸; Šečkutė & Deveraj *et al.*, 2013 ▸; Churakov *et al.*, 2004 ▸) as well as bridging ligands in metal complexes (Kaim, 2002 ▸; Clavier & Audebert, 2010 ▸). Moreover, their derivatives are often among biologically active compounds (Saghatforoush *et al.*, 2016 ▸) and play an important role in anti-inflammatory (Kamal *et al.*, 2006 ▸), anti­cancer, anti­viral drugs (Rao & Hu, 2006 ▸; Neunhoeffer *et al.*, 1984 ▸) or as insecticidal products (Sauer *et al.*,1996 ▸; Brooker *et al.*, 1987 ▸).
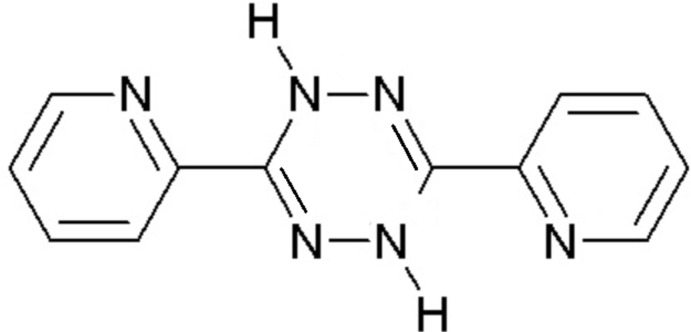


**Figure 1:**
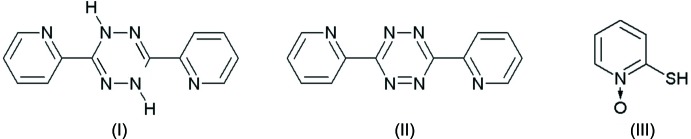
Mol­ecular formulae of: 3,6-bis­(pyridin-2-yl)-1,4-di­hydro-1,2,4,5-tetra­zine (I), 3,6-bis­(pyridin-2-yl)-1,2,4,5-tetra­zine (II) and 2-mercapto­pyridine *N*-oxide (III).

The title compound 3,6-bis­(pyridin-2-yl)-1,4-di­hydro-1,2,4,5-tetra­zine (I) was obtained as a yellow solid by reduction of 3,6-bis­(pyridin-2-yl)-1,2,4,5-tetra­zine (II) during its crystallization with 2-mercapto­pyridine *N*-oxide (III) in ethanol solution (Fig. 1 ▸).

## Structural commentary   

**Figure 2:**
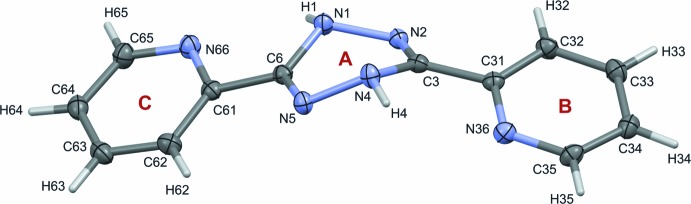
The mol­ecular structure of (I), showing the atom-labelling scheme and displacement ellipsoids at the 50% probability level.

Compound (I) crystallizes in the monoclinic space group *P*2_1_/*n*. The atomic labelling scheme is shown in Fig. 2 ▸. In (I), being a reduced form of (II), there are two hydrogen atoms at the 1 and 4 positions and two 2-pyridyl substituents at the 3 and 6 positions.

The C—C bond lengths are within the expected values known for aromatic systems (Allen *et al.*, 1987 ▸). However, there is a fluctuation of bond distances involving nitro­gen atoms. The N—N bonds within the central (*A*) ring are of almost equal length, being 1.4285 (15) and 1.4306 (16) Å. The C6—N1 and C3—N4 [1.3953 (17) and 1.4051 (17) Å] bond lengths are longer than those for C6—N5 and C3—N2 [1.2848 (17) Å, 1.2809 (18) Å], respectively. This is the result of the protonation of the N1 and N4 atoms. The C—N bond lengths in the *B* and *C* rings are comparable within 3σ, varying from 1.3384 (18) Å to 1.3416 (17) Å.

**Figure 3:**
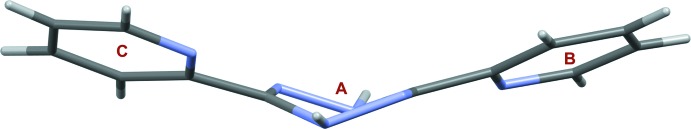
The butterfly-like mol­ecular conformation of (I).

The central tetra­zine ring (*A*) shows a boat conformation with pseudo-symmetry mirror planes passing through bonds N2—C3 and N5—C6 [ΔC_s_ = 1.30 (16)°] and atoms N1, N4 [ΔC_s_ = 2.00 (14)°]. In this conformation, hydrogen atoms are located in the equatorial positions of the ring and the N—H bonds are directed to the bottom of the boat (compare torsion angles in Table 1 ▸). The planes of the aromatic pirydyl rings (*B* and *C*) are not to parallel to each other. The dihedral angles between these rings and central tetra­zine ring are 22.43 (7)° (*A* and *B*) and 25.71 (6)° (*A* and *C*). The dihedral angle between rings *B* and *C* is 27.13 (7)°. The overall mol­ecular structure could be recognized as a butterfly-like conformation as shown in Fig. 3 ▸.

## Supra­molecular features   

**Figure 4:**
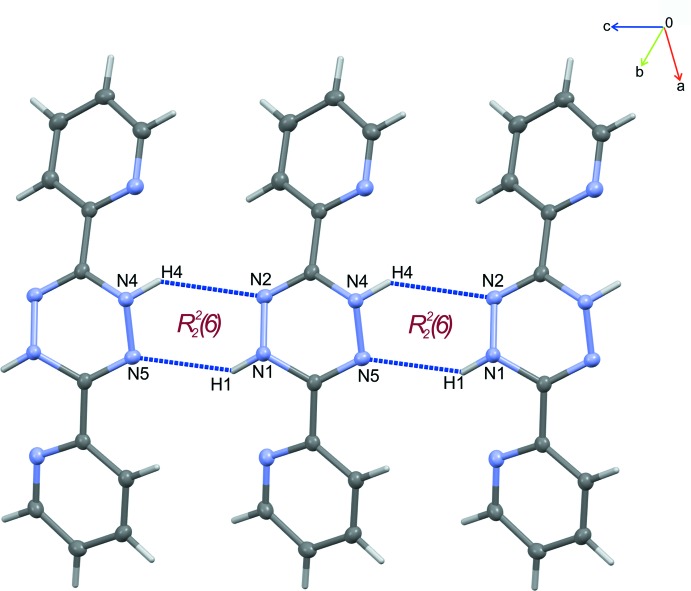
N—H ⋯ N hydrogen bonds between rings of 1,2,4,5-tetra­zine of adjacent mol­ecules forming a chain of cyclic dimers.

**Figure 5:**
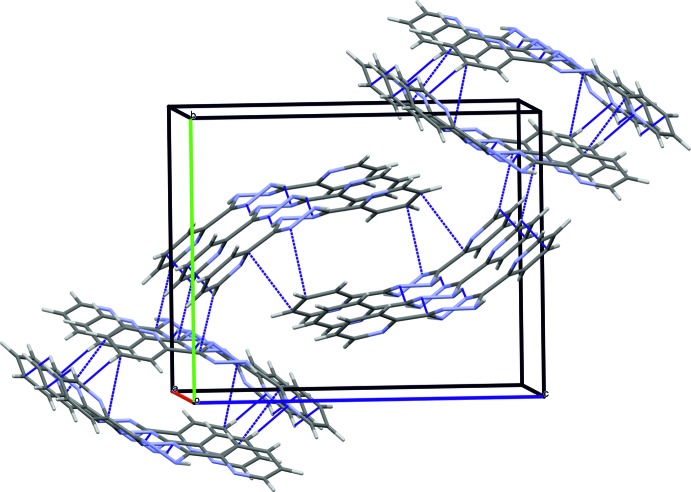
A view of the unit-cell packing, showing the ribbon-like arrangement of mol­ecules. Short C⋯N and C⋯C inter­molecular contacts between adjacent mol­ecular ribbons are shown as dashed blue lines.

The crystal packing of (I) is mainly determined by inter­molecular hydrogen bonds of the N—H⋯N type (Table 2 ▸). Firstly, two similar hydrogen bonds (N1—H1⋯N5 and N4—H4⋯N2) between the 1,2,4,5-tetra­zine rings of neighbouring mol­ecules form a chain with an *R* ^2^_2_(6) ring motif (Etter *et al.*, 1990 ▸) (see Fig. 4 ▸). As a result, the mol­ecules are ordered into infinite ribbons extending along the [100] direction. This parallel arrangement of the ribbons is additionally stabilized by further inter­actions between adjacent mol­ecules [N5⋯C33(1 − *x*, 1 − *y*, 1 − *z*) = 3.2418 (18) Å and C34⋯C61(1 − *x*, 1 − *y*, 1 − *z*) = 3.3334 (19) Å], as shown in Fig. 5 ▸.

## Database survey   

A search of the Cambridge Structure Database (CSD version 5.39, update of February 2018; Groom *et al.*, 2016 ▸) results in 76 deriv­atives of 3,6-bis­(pyridin-2-yl)-1,2,4,5-tetra­zine, among them compound (II) (refcode JUMXAQ; Klein *et al.*, 1998 ▸), which is the oxidated form of (I). Even tought (II) crystallizes in the smae monoclinic space group as (I), its molecular and crystal structures show completely different features.

## Synthesis and crystallization   

Crystals suitable for X-ray measurements were obtained from a commercially available reagent (Aldrich Chemical Co.) and used without further purification. 0.5 mmol of 3,6-bis­(pyridin-2-yl)-1,2,4,5-tetra­zine and 0.5 mmol of 2-mercapto­pyridine *N*-oxide (in a 1:1 molar ratio) were mixed in ethanol (4 ml). The resulting solution was warmed to 343 K and then kept at room temperature. Within two weeks, after slow evaporation of the solvent, two kinds of crystal were obtained in a crystallizer. X-ray studies confirmed that the pink crystals were of the known structure (II), while the yellow crystals were identified as being of a previously unreported structure, *i.e.* (I).

## Refinement   

Crystal data, data collection and structure refinement details are summarized in Table 3 ▸. Hydrogen atoms of aromatic rings were introduced in calculated positions with idealized geometry and constrained using a rigid body model with isotropic displacement parameters equal to 1.2 the equivalent displacement parameters of the parent atoms. The H atoms of the NH groups, in 1,2,4,5-tetra­zine ring, were located in a difference Fourier map and freely refined.

## Supplementary Material

Crystal structure: contains datablock(s) I. DOI: 10.1107/S205698901801753X/ff2157sup1.cif

Structure factors: contains datablock(s) I. DOI: 10.1107/S205698901801753X/ff2157Isup2.hkl

Supporting information file. DOI: 10.1107/S205698901801753X/ff2157Isup3.cml

CCDC reference: 1884403

Additional supporting information:  crystallographic information; 3D view; checkCIF report

## Figures and Tables

**Table 1:** Selected torsion angles (°)

N2—C3—N4—H4	164.1 (13)	C3—N2—N1—H1	−168.4 (12)
C6—N5—N4—H4	−165.2 (14)	N5—C6—N1—H1	164.3 (13)

**Table 2:** Hydrogen-bond geometry (Å, °)

*D*—H⋯*A*	*D*—H	H⋯*A*	*D*⋯*A*	*D*—H⋯*A*
N4—H4⋯N2^i^	0.89 (2)	2.56 (2)	3.3017 (16)	142.5 (17)
N1—H1⋯N5^ii^	0.880 (17)	2.415 (17)	3.1321 (16)	138.9 (15)

Symmetry codes: (i) 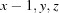; (ii) 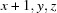.

**Table 3:** Experimental details

Crystal data	
Chemical formula	C_12_H_10_N_6_
*M*_r_	238.26
Crystal system, space group	Monoclinic, *P*2_1_/*n*
Temperature (K)	100
*a*, *b*, *c* (Å)	5.4603 (1), 12.7845 (3), 15.6474 (4)
β (°)	97.281 (2)
*V* (Å^3^)	1083.49 (4)
*Z*	4
Radiation type	Cu *K*α
μ (mm^−1^)	0.78
Crystal size (mm)	0.11 × 0.10 × 0.08

Data collection	
Diffractometer	Rigaku Oxford Diffraction SuperNova, Dual, Cu at zero, Atlas
Absorption correction	Multi-scan (*CrysAlis PRO*; Rigaku OD, 2015 ▸)
*T*_min_, *T*_max_	0.958, 1.000
No. of measured, independent and observed [*I* > 2σ(*I*)] reflections	8686, 2004, 1767
*R*_int_	0.027
(sin θ/λ)_max_ (Å^−1^)	0.603

Refinement	
*R*[*F* ^2^ > 2σ(*F* ^2^)], *wR*(*F* ^2^), *S*	0.035, 0.095, 1.12
No. of reflections	2004
No. of parameters	171
H-atom treatment	H atoms treated by a mixture of independent and constrained refinement
Δρ_max_, Δρ_min_ (e Å^−3^)	0.14, −0.24

Computer programs: *CrysAlis PRO* (Rigaku OD, 2015 ▸), *SHELXT2014* (Sheldrick, 2015*a* ▸), *SHELXL2014* (Sheldrick, 2015*b* ▸), *WinGX* (Farrugia, 2012 ▸), *PLATON* (Spek, 2009 ▸) and *publCIF* (Westrip, 2010 ▸).

## supplementary crystallographic information

### Crystal data

**Table:**

C_12_H_10_N_6_	*F*(000) = 496
*M**_r_* = 238.26	*D*_x_ = 1.461 Mg m^−^^3^
Monoclinic, *P*2_1_/*n*	Cu *K*α radiation, λ = 1.54184 Å
*a* = 5.4603 (1) Å	Cell parameters from 3734 reflections
*b* = 12.7845 (3) Å	θ = 4.5–76.4°
*c* = 15.6474 (4) Å	µ = 0.78 mm^−^^1^
β = 97.281 (2)°	*T* = 100 K
*V* = 1083.49 (4) Å^3^	Plate, yellow
*Z* = 4	0.11 × 0.10 × 0.08 mm

### Data collection

**Table:**

Rigaku Oxford Diffraction SuperNova, Dual, Cu at zero, Atlas diffractometer	2004 independent reflections
Radiation source: micro-focus sealed X-ray tube, SuperNova (Cu) X-ray Source	1767 reflections with *I* > 2σ(*I*)
Detector resolution: 10.4052 pixels mm^-1^	*R*_int_ = 0.027
ω scans	θ_max_ = 68.5°, θ_min_ = 4.5°
Absorption correction: multi-scan (CrysAlisPRO; Rigaku OD, 2015)	*h* = −6→6
*T*_min_ = 0.958, *T*_max_ = 1.000	*k* = −15→14
8686 measured reflections	*l* = −18→17

### Refinement

**Table:**

Refinement on *F*^2^	0 restraints
Least-squares matrix: full	Hydrogen site location: mixed
*R*[*F*^2^ > 2σ(*F*^2^)] = 0.035	H atoms treated by a mixture of independent and constrained refinement
*wR*(*F*^2^) = 0.095	*w* = 1/[σ^2^(*F*_o_^2^) + (0.051*P*)^2^ + 0.2596*P*] where *P* = (*F*_o_^2^ + 2*F*_c_^2^)/3
*S* = 1.12	(Δ/σ)_max_ < 0.001
2004 reflections	Δρ_max_ = 0.14 e Å^−^^3^
171 parameters	Δρ_min_ = −0.24 e Å^−^^3^

### Special details

**Table:**

Geometry. All esds (except the esd in the dihedral angle between two l.s. planes) are estimated using the full covariance matrix. The cell esds are taken into account individually in the estimation of esds in distances, angles and torsion angles; correlations between esds in cell parameters are only used when they are defined by crystal symmetry. An approximate (isotropic) treatment of cell esds is used for estimating esds involving l.s. planes.

### Fractional atomic coordinates and isotropic or equivalent isotropic displacement parameters (Å^2^)

**Table:**

	*x*	*y*	*z*	*U*_iso_*/*U*_eq_	
N5	0.3304 (2)	0.59460 (9)	0.30162 (7)	0.0159 (3)	
N1	0.7587 (2)	0.61063 (9)	0.30346 (7)	0.0167 (3)	
N66	0.7195 (2)	0.45247 (9)	0.18218 (7)	0.0178 (3)	
N4	0.3800 (2)	0.66133 (9)	0.37517 (7)	0.0162 (3)	
N2	0.7969 (2)	0.61146 (9)	0.39548 (7)	0.0166 (3)	
N36	0.4117 (2)	0.70258 (9)	0.54575 (7)	0.0196 (3)	
C3	0.6017 (2)	0.63816 (10)	0.42759 (9)	0.0151 (3)	
C31	0.6094 (2)	0.65389 (10)	0.52161 (8)	0.0159 (3)	
C6	0.5274 (2)	0.57389 (10)	0.26787 (8)	0.0150 (3)	
C61	0.5133 (2)	0.50651 (10)	0.19059 (8)	0.0153 (3)	
C62	0.2981 (2)	0.49884 (11)	0.13279 (8)	0.0183 (3)	
H62	0.1608	0.5397	0.1394	0.022*	
C65	0.7096 (2)	0.38393 (10)	0.11717 (9)	0.0191 (3)	
H65	0.8497	0.3444	0.1116	0.023*	
C64	0.5020 (3)	0.36871 (11)	0.05778 (9)	0.0194 (3)	
H64	0.5020	0.3195	0.0140	0.023*	
C34	0.6135 (3)	0.69920 (11)	0.69170 (9)	0.0201 (3)	
H34	0.6106	0.7167	0.7493	0.024*	
C32	0.8134 (3)	0.62397 (11)	0.57895 (9)	0.0196 (3)	
H32	0.9460	0.5895	0.5596	0.024*	
C63	0.2943 (3)	0.42869 (11)	0.06517 (9)	0.0201 (3)	
H63	0.1539	0.4219	0.0252	0.024*	
C33	0.8137 (3)	0.64675 (11)	0.66537 (9)	0.0215 (3)	
H33	0.9465	0.6272	0.7054	0.026*	
C35	0.4179 (3)	0.72475 (11)	0.62978 (9)	0.0211 (3)	
H35	0.2832	0.7593	0.6476	0.025*	
H1	0.886 (3)	0.5796 (14)	0.2850 (11)	0.022 (4)*	
H4	0.253 (4)	0.6611 (15)	0.4051 (13)	0.030 (5)*	

### Atomic displacement parameters (Å^2^)

**Table:**

	*U*^11^	*U*^22^	*U*^33^	*U*^12^	*U*^13^	*U*^23^
N5	0.0158 (5)	0.0169 (5)	0.0144 (5)	0.0008 (4)	−0.0001 (4)	−0.0012 (4)
N1	0.0134 (5)	0.0221 (6)	0.0148 (5)	−0.0014 (5)	0.0020 (4)	−0.0020 (4)
N66	0.0154 (5)	0.0182 (6)	0.0198 (6)	0.0000 (4)	0.0025 (4)	−0.0009 (4)
N4	0.0138 (5)	0.0196 (6)	0.0150 (6)	0.0025 (4)	0.0007 (4)	−0.0025 (4)
N2	0.0159 (5)	0.0191 (6)	0.0145 (5)	−0.0009 (4)	0.0003 (4)	−0.0013 (4)
N36	0.0174 (6)	0.0229 (6)	0.0181 (6)	0.0010 (4)	0.0009 (4)	−0.0024 (4)
C3	0.0138 (6)	0.0136 (6)	0.0173 (7)	−0.0002 (5)	−0.0001 (5)	0.0003 (5)
C31	0.0161 (6)	0.0144 (6)	0.0170 (7)	−0.0025 (5)	0.0015 (5)	0.0009 (5)
C6	0.0137 (6)	0.0149 (6)	0.0162 (6)	0.0006 (5)	0.0007 (5)	0.0022 (5)
C61	0.0151 (6)	0.0144 (6)	0.0166 (6)	−0.0010 (5)	0.0032 (5)	0.0015 (5)
C62	0.0153 (6)	0.0211 (7)	0.0185 (7)	0.0017 (5)	0.0018 (5)	0.0005 (5)
C65	0.0168 (6)	0.0175 (6)	0.0236 (7)	0.0007 (5)	0.0055 (5)	−0.0018 (5)
C64	0.0223 (7)	0.0183 (6)	0.0181 (7)	−0.0027 (5)	0.0049 (5)	−0.0025 (5)
C34	0.0253 (7)	0.0193 (7)	0.0155 (6)	−0.0053 (5)	0.0017 (5)	−0.0006 (5)
C32	0.0180 (7)	0.0204 (7)	0.0203 (7)	0.0013 (5)	0.0019 (5)	0.0023 (5)
C63	0.0175 (6)	0.0237 (7)	0.0183 (7)	−0.0024 (5)	−0.0005 (5)	0.0002 (5)
C33	0.0212 (7)	0.0231 (7)	0.0190 (7)	−0.0019 (5)	−0.0024 (5)	0.0038 (5)
C35	0.0209 (7)	0.0226 (7)	0.0202 (7)	0.0000 (5)	0.0040 (5)	−0.0033 (5)

### Geometric parameters (Å, º)

**Table:**

N5—C6	1.2848 (17)	C61—C62	1.3926 (18)
N5—N4	1.4306 (16)	C62—C63	1.385 (2)
N1—C6	1.3953 (17)	C62—H62	0.9300
N1—N2	1.4285 (15)	C65—C64	1.386 (2)
N1—H1	0.880 (19)	C65—H65	0.9300
N66—C65	1.3384 (18)	C64—C63	1.386 (2)
N66—C61	1.3416 (17)	C64—H64	0.9300
N4—C3	1.4051 (17)	C34—C35	1.387 (2)
N4—H4	0.88 (2)	C34—C33	1.389 (2)
N2—C3	1.2809 (18)	C34—H34	0.9300
N36—C35	1.3412 (18)	C32—C33	1.383 (2)
N36—C31	1.3415 (18)	C32—H32	0.9300
C3—C31	1.4800 (18)	C63—H63	0.9300
C31—C32	1.3922 (19)	C33—H33	0.9300
C6—C61	1.4786 (18)	C35—H35	0.9300
			
C6—N5—N4	111.75 (11)	C63—C62—H62	120.9
C6—N1—N2	114.45 (10)	C61—C62—H62	120.9
C6—N1—H1	115.4 (12)	N66—C65—C64	123.53 (12)
N2—N1—H1	108.3 (12)	N66—C65—H65	118.2
C65—N66—C61	117.28 (12)	C64—C65—H65	118.2
C3—N4—N5	113.90 (10)	C65—C64—C63	118.36 (13)
C3—N4—H4	111.4 (13)	C65—C64—H64	120.8
N5—N4—H4	110.1 (13)	C63—C64—H64	120.8
C3—N2—N1	112.02 (11)	C35—C34—C33	118.16 (13)
C35—N36—C31	116.93 (12)	C35—C34—H34	120.9
N2—C3—N4	121.69 (12)	C33—C34—H34	120.9
N2—C3—C31	120.37 (12)	C33—C32—C31	118.30 (13)
N4—C3—C31	117.75 (12)	C33—C32—H32	120.9
N36—C31—C32	123.55 (12)	C31—C32—H32	120.9
N36—C31—C3	114.85 (12)	C62—C63—C64	119.26 (13)
C32—C31—C3	121.54 (12)	C62—C63—H63	120.4
N5—C6—N1	121.95 (12)	C64—C63—H63	120.4
N5—C6—C61	119.77 (12)	C32—C33—C34	119.21 (13)
N1—C6—C61	118.25 (11)	C32—C33—H33	120.4
N66—C61—C62	123.33 (12)	C34—C33—H33	120.4
N66—C61—C6	115.02 (11)	N36—C35—C34	123.82 (13)
C62—C61—C6	121.63 (12)	N36—C35—H35	118.1
C63—C62—C61	118.13 (12)	C34—C35—H35	118.1
			
N2—C3—N4—H4	164.1 (13)	C3—N2—N1—H1	−168.4 (12)
C6—N5—N4—H4	−165.2 (14)	N5—C6—N1—H1	164.3 (13)

### Hydrogen-bond geometry (Å, º)

**Table:**

*D*—H···*A*	*D*—H	H···*A*	*D*···*A*	*D*—H···*A*
N4—H4···N2^i^	0.89 (2)	2.56 (2)	3.3017 (16)	142.5 (17)
N1—H1···N5^ii^	0.880 (17)	2.415 (17)	3.1321 (16)	138.9 (15)

Symmetry codes: (i) *x*−1, *y*, *z*; (ii) *x*+1, *y*, *z*.
